# Teachers' Perspectives on the Acceptability and Feasibility of Wearable Technology to Inform School-Based Physical Activity Practices

**DOI:** 10.3389/fspor.2021.777105

**Published:** 2021-11-18

**Authors:** Georgina K. Wort, Gareth Wiltshire, Oliver Peacock, Simon Sebire, Andy Daly-Smith, Dylan Thompson

**Affiliations:** ^1^Department for Health, University of Bath, Bath, United Kingdom; ^2^School of Sport, Exercise and Health Sciences, Loughborough University, Loughborough, United Kingdom; ^3^School for Policy Studies, University of Bristol, Bristol, United Kingdom; ^4^Faculty of Health Studies, University of Bradford, Bradford, United Kingdom; ^5^Centre for Applied Education Research, Wolfson Centre for Applied Health Research, Bradford Royal Infirmary, Bradford, United Kingdom

**Keywords:** physical activity, wearable technologies, data, teachers' views, primary school, school-based practice

## Abstract

**Background:** Many children are not engaging in sufficient physical activity and there are substantial between-children physical activity inequalities. In addition to their primary role as educators, teachers are often regarded as being well-placed to make vital contributions to inclusive visions of physical activity promotion. With the dramatic increase in popularity of wearable technologies for physical activity promotion in recent years, there is a need to better understand teachers' perspectives about using such devices, and the data they produce, to support physical activity promotion in schools.

**Method:** Semi-structured interviews were conducted with 26 UK-based primary school teachers, exploring their responses to children's physical activity data and their views about using wearable technologies during the school day. Interview discussions were facilitated by an elicitation technique whereby participants were presented with graphs illustrating children's in-school physical activity obtained from secondary wearable technology data. Interview transcripts were thematically analyzed.

**Results:** Most teachers spoke positively about the use of wearable technologies specifically designed for school use, highlighting potential benefits and considerations. Many teachers were able to understand and critically interpret data showing unequal physical activity patterns both within-and between-schools. Being presented with the data prompted teachers to provide explanations about observable patterns, emotional reactions—particularly about inequalities—and express motivations to change the current situations in schools.

**Conclusion:** These findings suggest that primary school teachers in the UK are open to integrating wearable technology for measuring children's physical activity into their practices and can interpret the data produced by such devices. Visual representations of physical activity elicited strong responses and thus could be used when working with teachers as an effective trigger to inform school practices and policies seeking to address in-school physical inactivity and inequalities.

## Introduction

Physical activity is important for children's physical and mental well-being, cognitive and social development, and for providing strong foundations for future health, (Hansen et al., 2018; Wassenaar et al., 2020) and academic performance (Barbosa et al., 2020; Norris et al., 2020). Moreover, there are substantial disparities in children's physical activity, with several studies highlighting girls (Steene-Johannessen et al., 2020), and those from lower socio-economic backgrounds (Kay, 2016; Moore et al., 2017) are less active. Schools are often seen by a variety of stakeholders as promising settings in which to positively impact physical activity and wider health behaviors (Spotswood et al., 2019). Yet, previous school-based interventions have had limited success (Love et al., 2018; Cassar et al., 2019) and there is ongoing uncertainty about the most effective approaches to influence physical activity in schools (Daly-Smith et al., 2020a).

There has been a dramatic increase in the popularity and availability of wearable technologies in recent years, with products such as the *Fitbit* and the *Apple Watch* having significant commercial success. Wearable devices—and the data they produce—are now firmly established within what Millington (Millington, 2016) refers to as the second fitness boom. Amongst researchers seeking to promote physical activity, wearable technologies are increasingly used as a tool to support behavior change (Brickwood et al., 2019; Western et al., 2019). Several school-wide physical activity interventions have reported positive outcomes when using pedometers or wearable technologies for monitoring or support (Salmon et al., 2011; Eather et al., 2013; Morris et al., 2019). Indeed, it has been argued that data-driven decision making can inform and develop teachers' educational practices (Mandinach and Gummer, 2015). As wearable technologies, and their digital platforms continue to improve, it is becoming increasingly feasible to use these technologies to inform strategies, interventions, and whole school approaches to physical activity promotion. However, within this rapidly changing context there is an ongoing need to learn about how wearable technologies could be used in schools, understanding the perspectives of end-users, particularly regarding the potential risks.

Using technology in schools inevitably brings broader pedagogical and well-being concerns as schools are primarily places of learning and development. Whilst Borthwick et al. (2015) note the novelties and enhancements that technology can bring, the authors also raise concerns about privacy and security of data, reliance on private companies, and equality of access for all students. However, in contrast, there is optimism about the use of technology as evident in Casey et al. (2017) who argued that technology has “the potential to be an invaluable pedagogical device to support learning in individually and developmentally-appropriate ways” (p. 299). Furthermore, a key point made by Casey et al. (2017) is that a profession-wide debate about the use of technology in schools is needed and that teachers' perspectives are critically important.

Away from the discussion about the possible benefits and dangers of wearable technology to support school-based physical activity, several studies have focused on aspects of uptake and implementation. Marttinen et al. (2020) illustrate that when teachers do use wearable technology, they use it to augment—rather than replace—their existing practices and implement it within their chosen pedagogical approach. Bodsworth and Goodyear (2017) challenge the assumption that teachers are competent and confident using digital technology to support learning, highlighting the importance of reflexivity to refine and develop their practices. Wyant and Baek (2019) acknowledge that uptake of technology has been slow and make suggestions for better supporting teachers who wish to adopt it. Similarly, Almusawi et al. (2021) outline eight conditions that they found to impact teachers' readiness to integrate wearable technology; crucially, they highlighted the importance of teachers' positive appraisals about being able to track progress and to encourage movement through quantified monitoring.

The broad purpose of this study was to better understand teachers' perspectives about the use of wearable technologies in primary schools. This purpose is justified on the grounds that physical inactivity and related inequalities remain an ongoing challenge, and teachers often play a vital role in influencing children's physical activity (Eather et al., 2013; Daly-Smith et al., 2020a), with wearable technology increasingly being used within and outside of schools as a contemporary innovation. Indeed, as Morris et al. (2019) suggest, consulting and engaging with teachers is important when developing and implementing physical activity promotion strategies.

Taken together, the growing body of literature in this area suggests that, whilst critical concerns have been raised, wearable technology could have positive benefits for pupils. Moreover, it appears that teachers are interested in engaging in technologies despite there being important challenges to overcome. Our intention here is to contribute to this knowledge base by attempting to answer the following questions: (1) What do teachers believe to be the potential benefits and concerns about the use of wearable technologies within schools?, (2) How well do teachers understand visual representations of physical activity data from wearable technologies?, and (3) How do teachers respond to physical activity data from wearable technologies?

## Methods

This project received ethical approval from the Research Ethics Approval Committee for Health at the University of Bath (EP 19/20 046). Participant information sheets were provided online, and consent obtained, before arranging interviews at participants' convenience. Before being able to explore teachers' perspectives on the use of wearable technologies it was first necessary to collect and analyse children's in-school physical activity, producing a series of illustrative graphs. These graphical visualizations were then used in the empirical phase of this study as a basis for discussion within qualitative interviews.

### Physical Activity Data

It was important to ensure that the data visualizations being presented to teachers were derived from an authentic data set using reliable physical activity measures. Anonymised physical activity data was obtained from Moki Technology® (Moki Technology Ltd., 2021)^1^ from the 2019/20 academic school year, before school closures relating to COVID-19 (September 2019–February 2020). The mechanical reliability of these devices was explored using a Multi-Axis Simulation Table (MAST-9720; Instron Structural Testing Systems Ltd., High Wycombe, UK), (see Supplementary Material).

**Figure 1:**
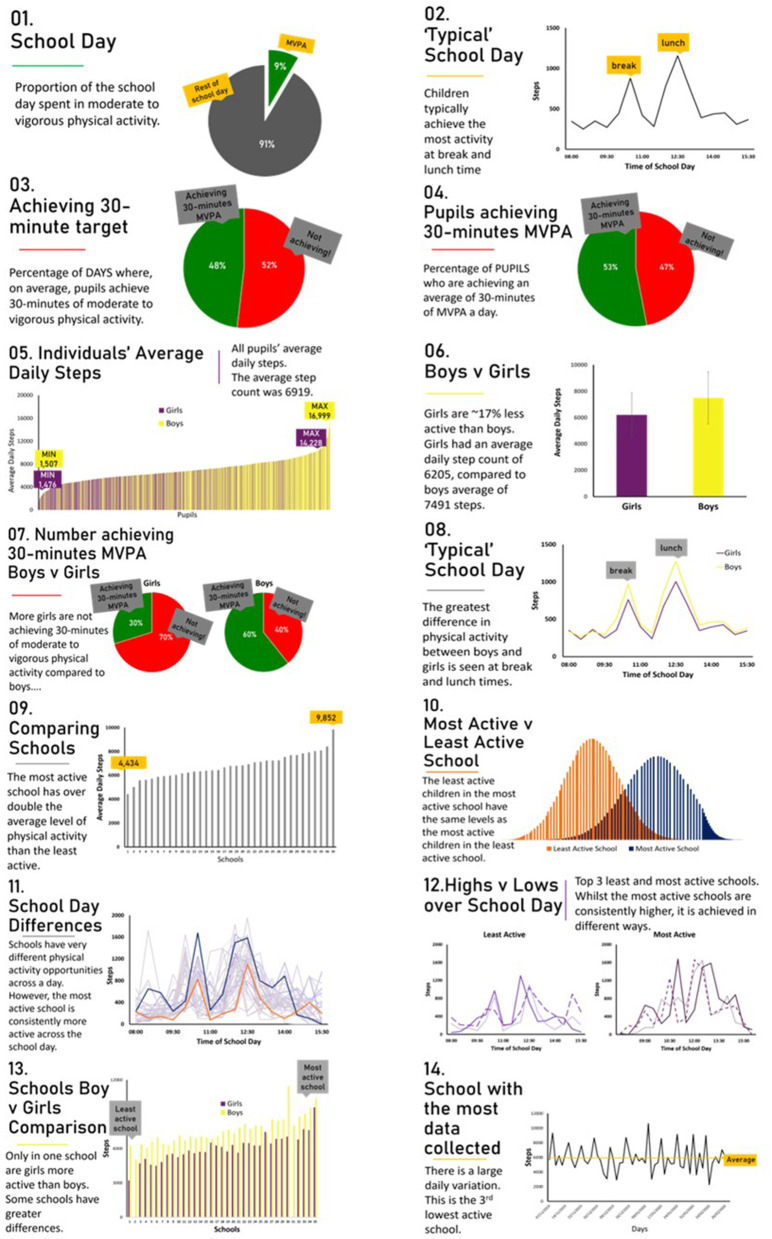
Graphs shown to teachers during semi-structured interviews. *Please note data in graph 4 should be presented the other way round, with 47% of pupils achieving MVPA target.

The dataset included 2,053 pupils. However, in line with previous research, pupils' data was included in the analysis if at least 3 school days had been collected (Jago et al., 2018; Daly-Smith et al., 2021), of more than 4 h between 8:00–16:00. A criterion of 30-min of zero counts was used to determine non-wear time and removed from the analysis. The final sample included 1,234 pupils (686 boys, 548 girls) from 35 schools. The average number of days collected was 12 (3–58 days, SD = 10), with an average of 6.5 h per day (4.5–8 h, SD = 0.5). Microsoft Excel was used for data cleaning, analysis, and to create 14 simple graphs displaying in-school physical activity within, and across, the school day (Figure 1). Graphs were produced from raw data in Microsoft Excel, not from Moki software. Graph descriptions are provided in Table 1.

**Table 1:** Graph descriptions.

**Graph number**	**Depiction**
1	The proportion of the school day pupils spent in MVPA
2	Physical activity (step counts) over the duration of in-school time
3	The percentage of school days meeting the 30-min per day MVPA target
4	The percentage of pupils meeting the 30-min per day MVPA target on average
5	All pupils' average daily step count
6	The average difference in step count between boys and girls
7	The percentage of pupils meeting the 30-min per day MVPA target on average split between boys and girls
8	Physical activity over the duration of in-school time split boys and girls
9	The average step counts across schools
10	A comparison of pupils from the least active and most active school (bell curve)
11	Physical activity over the duration of in-school split by individual schools
12	Physical activity over the duration of in-school split by the three most and three least active schools
13	The average step count across schools, split by boys and girls
14	One school's daily variation in step counts

### Qualitative Interviews

#### Participants

Primary school teachers from across the UK were recruited between July-August 2020. The aim was to recruit teachers representing different demographics and regions of the UK. The final sample included 26 teachers, from 23 schools, across 13 counties within the UK (Table 2). Three teachers had personally used Moki Technology® before, and three others had heard of the company. The data did not contain information regarding school identity. It is feasible that the three teachers who had used Moki devices previously had data from their schools included in the dataset. However, all data was anonymized and confidential, therefore, it was not possible for the teachers, nor the researchers, to know this or to comment on specific schools.

**Table 2:** Comparison of teachers interviewed within the sample.

**No**.	**Years teaching**	**Current teaching role and PA interest**	**Female/male**	**School description from teachers' perspectives**
1	4	Year 3 Class teacher	Female	Mixed affluent and deprived, high% FSM, 3 form entry. Urban.
2	31	Year 2 Class teacher, assistant head SENCo, computing and music lead	Female	Mixed affluent and deprived. 1 form entry. 208 pupils. Semi-rural.
3	29	Head teacher. Interest in PA	Male	6% FSM, 1 form entry. Semi-rural.
4	8	Senior Leader. Sport and well-being lead.	Female	24% PP, 17% FSM. 55% EAL 419 pupils. Urban.
5	5	PE coordinator, only teaches PE.	Female	Mixed affluent and deprived. 1 form entry. Low% PP. Semi-rural.
6	4	SEND teachers	Female	Special needs school, 206 pupils aged 5–16 years. Semi-rural.
7	15	Year 4 Class teacher. Head of science.	Female	8% FSM, 14% PP 210 pupils, 1 form entry. Semi-rural.
8	4	Year 2 class teacher	Female	Independent school. 330 pupils. Urban.
9	34	Deputy head, Year 4/5 job share. PE lead (10 years)	Female	11% FSM. 22% EAL, 260 pupils Semi-rural.
10	13	Year 3 class teacher	Female	Mixed affluent and deprived, high number with “social issues” 450 pupils, 2 form entry. Semi-rural.
11	3	Year 1 class teacher	Male	“Challenging, struggling families” 500–600 pupils, 2 form entry. Semi-rural
12	5	Year 6 class teacher and focus teacher	Female	30% FSM, 2 form entry. Semi-rural.
13	7	Year 5 class teacher, PSHE and language lead.	Female	Increasingly deprived, increasing FSM. 1 form entry. Semi-rural.
14	6	Year 2 class teacher, PE and maths coordinator, NQT mentor.	Male	Middle large estate. 2 form entry, over-subscribed, 180 pupils. Rural.
15	1	Year 4/5 class teacher, NQT year.	Female	250 pupils, 1/ 1.5 form entry. Rural.
16	27	Supply teacher in infant's several supply roles over years.	Male	Low% FSM. 230 pupils. Urban
17	10	Year 6 Class teacher, head outdoor learning	Male	Independent school, 256 children, 2 form. Semi-rural.
18	10	PE-coordinator only teaches PE. Secondary school background for 3 years.	Female	Relatively high FSM, 2 form entry, 470 pupils. Urban.
19	4	Year 6 Class teacher. Head KS2.	Female	Mixed affluent and deprived, 407 pupils, 2 form entry. Urban
20	11	Head of sport	Male	Independent school, 256 children, 2 form. Semi-rural
21	6	Year 3 class teacher, music coordinator.	Female	Middle class area, 2 form entry. Rural.
22	8	Head of girls' games	Female	Independent school, 256 children, 2 form. Semi-rural.
23	20	Year 6 class teacher, Head of Year 6, Healthy school coordinator (previous secondary background, changed 3 years ago)	Female	Low socio-economic area, ~31% FSM. 480 pupils, 4 form entry. Semi-rural.
24	5	Year 6 Class teacher	Female	Low socio-economic area, ~31% FSM. 480 pupils, 4 form entry. Semi-rural.
25	8	Year 1 class teacher, job share, SENCo	Female	8.9% FSM. 3 form entry, >600 children. Urban.
26	5	Head of girls' games	Female	Independent school, 256 children, 2 form. Urban.

*FSM, free school meals; EAL, English as an additional language; PP, pupil premium; SENCo, Special Educational Needs Co-ordinator*.

#### Data Collection

Semi-structured interviews were conducted online and recorded using Microsoft Teams due to COVID-19 restrictions, with attention paid to recent guidance about conducting qualitative research during a pandemic (Marhefka et al., 2020). The mean interview time was 65 min (51–83 min, SD = 7.5). Interviews were conducted by the lead author, and were used to facilitate in-depth discussion, allowing participants' views, interpretations, and experiences to be obtained. Interviews were structured around the research questions for this study but were applied flexibly to account for the possibility of new directions and to accommodate diversity in what individual participants believed to be important. At the start of each interview, the Moki devices were described to teachers and an explanation was provided regarding how the data was obtained to create the graphs. Initial questions were asked about teachers' backgrounds, views of children's physical activity (e.g., what do you think the barriers are to children's physical activity?), and schools' specific influence on physical activity (e.g., how do you think schools encourage physical activity?). Subsequently, the graphs shown in Figure 1 were introduced sequentially and teachers were asked for their opinions on the information presented. Teachers did not have access to the graphs prior to interviews and were shown one slide at a time, through the “share screen” feature of Microsoft Teams. Presenting the data during the interviews in this way allowed participants' immediate thoughts and feelings about physical activity graphs to be recorded. The purpose of showing the graphs to teachers was to investigate their understanding of graphical illustrations depicting pupils' physical activity within schools, as obtained from wearable technologies, and to explore how they responded to information portrayed in this manner. Finally, teachers were asked about the use of wearable technology within schools. Interviews were audio recorded and notes made after each interview to allow the lead author to record detailed initial perceptions of the interview, which were later drawn on during the data analysis stage. This process also helped facilitate personal reflectivity and the lead author acknowledges that their position as an early-career researcher and “outsider” (having not taught in schools) likely had a significant impact on how the interviews were conducted and analyzed. This positionality allowed the lead researcher to take an impartial and naive approach to interviewing. An awareness of this helped guide the subsequent analysis and discussions with co-authors.

#### Data Analysis

Interviews were transcribed verbatim by the lead author shortly after conducting the interviews. Not only did this help to reduce threats to descriptive validity (Maxwell, 2012), but it also had the benefit of encouraging researcher reflections between interviews, increasing familiarity with the data and initiating early analytic thinking. False starts and irrelevancies (such as “ums,” repeated words, or incomplete sentences) were omitted from the transcripts, unless it added meaning, and any personal or identifiable information was deleted to preserve anonymity.

Transcripts were analyzed within NVivo 12, broadly aligning to Braun and Clarke (2006) six-step thematic analysis process, following more updated guidance regarding reflective thematic analysis (Braun and Clarke, 2019; Finlay, 2021; Wiltshire and Ronkainen, 2021). Coding was initially data-driven, giving primacy to the development of themes with meanings that originated in participant perspectives. Categorizing data then shifted to a question-driven approach (i.e., which research question does this data help answer?). These were iterated several times—sometimes in response to discussions within the research team—until the lead author was satisfied with the extent to which themes were a reasonable reflection of the empirical data. In line with recent recommendations in thematic analysis it was considered important to record and report how many of the 26 participants the various themes could be attributed to (Wiltshire and Ronkainen, 2021).

## Results

### Perspectives on Wearable Technologies

The majority (*n* = 23) of teachers spoke positively about the use of wearable technologies specifically designed for schools. For example, one participant said, “*it's more positive than anything; I think it would be a good way to track and trace” (Teacher 11)* and another said, “*I would really like to be able to introduce them within schools” (Teacher 23)*. When further explaining these positive evaluations, participants described a range of potential benefits. For most teachers (*n* = 21), the devices could provide valuable information:

*I thought that would be a really good thing to do because I like to have data. I like to be able to say to the teachers, “this is why we're doing this” and “this is what we want to achieve.” (Teacher 4)*

Similarly, many participants (*n* = 18) thought that data could be used to identify children with lower levels of activity and illuminate periods of inactivity. For example, one teacher thought that wearable technology data “would definitely make me as a teacher realize, ‘*okay so on Mondays we don't have this activity, let's make sure we do this.”' (Teacher 19)*

Teachers thought that activity bands would impact pupils in various direct ways, believing that the bands would be stimulating and enjoyable (e.g., “*I think the kids would love it”—Teacher 1*), or would increase motivation because pupils would have a new sense of what “enough” physical activity means (e.g., “*some of them would be like, “oh I haven't done enough steps today” and would run around to get their steps up”—Teacher 1*), or become competitive (e.g., “*it will become a competition because somebody else has done more steps than others, which is good in some respects, if it's done in the right way”—Teacher 25*). Several participants (*n* = 10) suggested that the bands could be effective if used as part of individual or team goal-setting activities (e.g., “*if targets were set that children found manageable then that could be a motivator”—Teacher 13*). A minority of participants (*n* = 3) also suggested that the activity bands could aid classroom learning. As one teacher said, “*I could see the children using their own data from a week to then plot graphs in maths … they'd really love that, because they like to use themselves for data.” (Teacher 19)*

Teachers however, placed caveats around some of the potential benefits discussed. Several participants (*n* = 9) believed that teachers' support and involvement in wearing activity bands would be important for successful integration. For example, one teacher thought that “*children would buy into it if staff also bought into it” (Teacher 24) and another asserted that “if the children are wearing them, the staff need to wear them”* (Teacher 12). Teachers explained that compared to other wearable devices on the market, those without a screen, with the capacity for teachers to manage and involve all pupils, would reduce concerns relating to class distractions, unequal access to devices, development of obsessive behaviors and negative competition among pupils. The following two examples illustrate these perspectives:

…*the fact that it can be directed by teachers, so it could be determined how it was used–how often you want to feedback steps or how often you don't; and whether that's on an individual basis or just as a class report–then that would be positive. (Teacher 24)*

*I feel that if everyone in the school, or class, had them, then that would be better than, for example, using their [branded device]. Because obviously everyone can't access [branded devices] or watches like it. (Teacher 15)*

Teachers raised important concerns; the most widely held, was that the activity bands could be a “fad” or “novelty.” As one teacher put it, “*I think the concept is a great idea … my only qualm with it would be, would it have longevity?” (Teacher 18)*. Even in the short-term, some (*n* = 6) showed skepticism about accuracy and reliability (e.g., “*I'm not against them or anything like that but I do wonder how accurate they are”—Teacher 25*). Irrespective of accuracy, teachers (*n* = 7) commented that children may lack the understanding of step counts, and why data is being collected (e.g., “*I don't know how they use them, whether they actually understand the meaning of how many steps they've done, or whether they just have them as an accessory”—Teacher 22)*.

Concerns over the additional time and responsibility burden on teachers was talked about by almost half of those interviewed. Participants (*n* = 7) worried that using activity bands could make teaching more challenging if they caused disruption. As one participant said, “*just from a physical band perspective, [they] would need to be interesting enough for a child to wear, but not distracting. Because otherwise I'd have a collection of them on my desk!” (Teacher 13)*. Moreover, teachers (*n* = 10) suggested that the cost of devices would be an issue for schools (e.g., “*I think the cost implication is huge to the schools. Budgets are tight”—Teacher 12*) which was important because some teachers anticipated that children “*will lose them and break them” (Teacher 6)*.

Some teachers expressed concerns about the potential adverse effects on children themselves. Nine participants expressed discomfort about controlling, monitoring, or policing physical activity in schools (e.g., “*we can promote, and we can encourage, but I don't think we should police”—Teacher 2*). This discomfort was related to fears about the development of obsessive behaviors (e.g., “*my only concern would be instilling obsessive behavior at a young age, you know … that obsessive counting”—Teacher 1*) as well as the harmful impacts of social comparison and competitiveness (e.g., “*it could be demotivating if it suddenly highlighted to you how few steps you're doing compared to somebody else”—Teacher 17*). A few also mentioned minor concerns about the health and safety risks of wearing bands during physical education because they could be a “*catching risk” (Teacher 5)*.

### Comprehension of Physical Activity Graphs

The teachers' responses established confidence that the graphs effectively communicated clear physical activity messages and were pitched at the appropriate level. For example, when presented with Graph 5, illustrating the range in pupils' step count during school hours, one participant said:

*Gosh who was that lad doing about 16,000 steps a day? And then it goes right down to, what's the lowest, they're about 2,000, oh minimum 1,500 oh I see their number. God, what a difference between two children there! (Graph 5, Teacher 16)*

Similarly, when presented with Graph 10, illustrating the intersect of pupils from the most and least active school in the sample, a different participant responded:

*Wow. So, in your setting you might think that you're doing really well, but then your most active children are only doing stuff [physical activity] at the same [level] as those at the least. Or you can put it the other way, can't you, well our least active are at least doing the same as some children who are the most active. (Graph 10, Teacher 9)*

Whilst some teachers believed data regarding their school would be different across some of the graph metrics, no teacher explicitly disputed the information depicted. Some teachers (*n* = 6) were more critical in their understanding, acknowledging that MVPA is a more challenging intensity to reach, and that certain activities would be below this threshold, despite being of value:

*If they're climbing up a fort or a rope ladder, it may not be vigorous, but actually for their coordination and motor-skills, that's quite an important thing if they were doing it over and over and all throughout the afternoon. (Teacher 20)*

Similarly, 11 teachers in this study commented on data collection considerations, such as time of year, and how monitors were used (i.e., as a measurement or to motivate physical activity), indicating a deeper level of scrutiny about the data. Teacher 13, said “*using things like the wearable tech to carry out studies, I guess you kind of want them to use it in the same way.”* Indeed, there were also comments about either sensitivity, reliability, or reactivity when using activity trackers:

*It depends on what the trackers are picking up in terms of movement … the sensitivity of that tracker. (Teacher 3)* *. the fact that they might not keep it on the whole time and then I don't know how that would affect the data. (Teacher 21)*

None of the 26 teachers in this study misunderstood, or needed further explanation of graphs 1, 2, 7, 8, 9 and 13. However, a proportion (*n* = 9) found at least one of the graphs challenging to understand and required clarification. These challenges included requiring further explanation of MVPA (graph 1) and difficulty differentiating between graph 3 and 4 (i.e., discussing individual pupils meeting 30-min of MVPA instead of the number of school days the target was met). Furthermore, there were also difficulties for some teachers in understanding a bell curve (graph 10), (e.g., “*sorry I'm just trying to understand, I've never seen a bell curve before”—Teacher, 21*) or finding the multiple lines plotted on a single graph confusing (graph 11 and 12), (e.g., “*I mean I find these ones [graphs] a bit tricky to interpret”—Teacher 22*).

### Responses to Physical Activity Graphs

Broadly, we observed three kinds of responses to the graphs: (i) those that offered explanations to the data; (ii) responses that were emotional, involving feelings of sadness, pity, or surprise, and (iii) responses that suggested a motivation to change the current situations in schools. Although we present these responses separately, within the transcripts they were often intertwined and overlapping as participants made sense of the data.

Responses that offered explanations of the data contained claims relating to policy, the physical environment, the social environments in and outside of school, opportunities, and pupil characteristics. Many participants used all these factors at some point to explain the data, suggesting a high degree of agreement on the determinants of physical activity. Policy-oriented explanations included government policies, academic pressures, and sedentary classroom learning. As *Teacher 14 explained: “I do think there's a lot of expectations from the government for the curriculum, there's a lot of content … and I can see why children are not moving very often.”* School uniform, particularly girls', was highlighted as a barrier, with some teachers acknowledging that it needed to be changed to be “*fit for an active day” (Teacher 5)*. Participants also rationalized that the physical environment was also a contributing factor, highlighting the importance of space, school structure, weather, and equipment. Space was an important factor when discussing the gender disparities; teachers highlighted that football often dominated the playground space, limiting girls' ability to engage in other physical activities. For example:

*We have a massive playground and then there are other schools who have a playground the size of a postage stamp and even if those kids wanted to be active, they couldn't be because you can't actually get out of breath running across their playground.” (Teacher 5)*

“*… otherwise they would just take over the whole field … and you'd look out and the whole field had boys running around playing football and all around the edges were the girls” (Teacher 9)*

All teachers acknowledged that the opportunities available to children would influence their physical activity. Examples spanned across break times, P.E., sports clubs, active travel, opportunities within the curriculum, and family/community opportunities. Some teachers felt that all children had the same opportunities within the school day, (i.e., “*… they both have the same opportunities in their day-to-day schooling hours.”—Teacher 8*), whilst others believed girls were disadvantaged, (i.e., “*What are we offering for our girls?”—Teacher 12*).

There was also wide agreement that the social environment in school, including teachers, playground supervisors, school leadership, and the school “ethos” were significant factors. This was highlighted by *Teacher 13 who remarked “They must have a really strong ethos around physical activity that is embedded across the whole school day.”* In terms of the social environment outside of school, parents, socio-economic status, and wider societal factors were used to explain low activity levels, either because of a lack of value placed on physical activity, or gender stereotypes which created additional barriers. As *Teacher 1 noted, “I think it's quite a deep-rooted problem in our society. A lot of activities that boys do is involving sports and running around and it's not really the same for girls.”*

All teachers in this study attributed some of the variation in activity levels to pupils' individual characteristics, such as competence, confidence, motivation, personal preferences, and innate differences. Teachers had conflicting opinions regarding whether gender differences were accountable to innate, physiological differences or were instead influenced by societal pressures. Examples of these include:

*I think about those children during my brain breaks, or during my daily mile, or during their P.E. lessons who have really low confidence, they have no invested interest in physical activity and don't feel like it's something that they do. (Teacher 19)*

*The boys are quite dominant. They're a lot more skilled than the girls would be if they tried to join in. It's a bit of a vicious cycle, cause the boys are better cause they play more. (Teacher 22)*

It is not our intention to assess the validity of these claims here, only to report that presenting physical activity data to teachers is likely to initiate these kinds of reflections.

The second kind of response we observed was emotional, involving feelings of shock, sadness, pity, and surprise about the observed patterns (*n* = 22). Expressions of shock were evident after seeing graphs depicting low levels of MVPA, small proportions of children meeting guidelines, or differences between groups or schools. *Teacher 4 said “I'm quite shocked by that. That's not very good at all”* (Graph 1) and *Teacher 18 said “It's a large proportion of girls that are not . Bloody hell”* (Graph 7). Noticing the gender differences in physical activity brought about a feeling of disgust for Teacher 14 who said, “*the rest of the boys are a high proportion more active than girls on all of them, especially number 30. That's quite disgusting”* (Graph 13). Sadness was expressed when graphs depicted individual, gender, or school disparities. For example, “*you kind of know that it is true. It's just sad” (Graph 5, Teacher 1), or “you look at that orange line and feel sorry for those kids who don't get those opportunities either.” (Graph 11, Teacher 16)*

The third kind of response observed were those that suggested a motivation to change the current situations in schools. For some teachers (*n* = 10), exposure to new information not only resulted in increased awareness but, crucially, seemed to trigger feelings of being enlightened. Feelings of this nature can be seen in responses like, “*well until we were talking about it now, I haven't really thought about it being an issue” (Graph 2, Teacher 13)*. Some responses suggested that participants might have shifted toward a belief that physical activity ought to be taken more seriously. Examples included:

*From those 35 schools it definitely shows that there's an issue that should be looked at.” (Graph 13, Teacher 7)*

*With data like this you start to think along different lines because at the moment it's very much the status quo is fine, they [children] all seem to be quite happy, but actually when you see statistics like this then it's actually they [children] might be quite happy doing what they're doing, but it's not necessarily the best thing for them. (Graph 7, Teacher 17)*

Something that often followed from these responses was an enthusiasm to be more proactive about promoting physical activity. Eleven of the teachers expressed sentiments about being more enthusiastic and proactive:

*Looking at all this data that you've got, it is scary and it's making me think actually, I need to do a bit. (Teacher 25)*

*I am not P.E. orientated, I have a passion for foundation, so it's making me really think about outdoor curriculum use and the positive impact on their physical activity levels. (Teacher 12)*

*It's definitely made me…think about movement a bit more, especially on those rainy days and especially on days where you don't have any activity. (Teacher 19)*

## Discussion

Our main aim was to better understand teachers' perceptions about the use of wearable technologies within schools and investigate how teachers understand and react to data visualizations derived from wearable technologies. Overall, the findings showed teachers believed that the use of wearable technologies, specifically designed for schools, was feasible and acceptable. We also found that physical activity data from wearable technologies can be used to communicate children's physical activity to teachers. Teachers were able to provide potential explanations for within-and between-school patterns and differences, and their responses to data included emotion, heightened awareness, and motivation to change the current situation in schools. Thus, these results highlight the promise data derived from wearable technologies has for informing teachers' and schools' understanding of pupils' physical activity, with the potential to influence school practices.

Overall, teachers were positive about the use of devices specifically-designed to measure physical activity within schools, believing they could be used to highlight inactive periods of the school day, or identify less active pupils, and ultimately positively impact pupils' physical activity. Previous research has demonstrated the benefits of wearable technologies for monitoring the fidelity and effectiveness of physical activity interventions (Salmon et al., 2011; Eather et al., 2013; Morris et al., 2019; Mavilidi et al., 2020) and our findings support the use of technologies for tracking progress and encouraging movement through quantified monitoring (Almusawi et al., 2021). Several teachers expressed interest in using the devices within their schools for these purposes. Whilst these findings suggest that wearable technologies could be used to empower teachers and schools, further research is required to investigate how teachers integrate devices into the school day. Insight is also needed into what role wearable monitors can play in supporting whole school approaches to increase children's physical activity and address inequalities over the long-term.

Teachers highlighted several positives related to the potential use of wearable technology in schools, such as increased motivation, goal setting, competition, and increased physical activity, which have been previously discussed within the literature (Brickwood et al., 2019). Teachers also highlighted the potential of these new technologies to benefit classroom learning, suggesting data could be incorporated into maths or science lessons. These findings support views that these technologies could benefit pupils' engagement in physical activity, support learning, and be implemented into wider school practices (Wyant and Baek, 2019). Teachers acknowledged the importance of children understanding why devices are being used, and the meaning of the data. Further research should be conducted to understand pupils' perspectives and their responses to these devices and data, investigating whether wearable technologies could be used to support pupils' learning, in addition to benefiting physical activity.

Whilst teachers were broadly positive about the use of technologies, it is important to acknowledge that they considered the possible negative implications of integrating these technologies within primary schools. These concerns included: fears of over monitoring, the novelty of devices reducing in the medium-long term, health and safety concerns, bands becoming broken or lost, and the cost of devices. Fears of teachers' surveillance over pupils' physical activity has been previously raised (Kerner et al., 2019) and this warrants consideration. Indeed, use of personal/individual Fitbit devices has been reported to decrease adolescence motivation and MVPA (Kerner et al., 2019). Previous research has also indicated that there could be negative health and well-being outcomes from self-tracking by encouraging obsessive monitoring behaviors and peer comparisons (Goodyear et al., 2019; Kerner et al., 2019; Lupton, 2021). Therefore, careful consideration should be given to the way in which these technologies are implemented. As acknowledged by the teachers in this study, key issues may be negated if the teacher is able to manage the data, rather than pupils self-tracking. Teachers also highlighted concerns about the potential for bands to generate classroom distractions, and negative competition between pupils, although they also highlighted that these concerns would be negated if there was no device screen and devices were managed by teachers. Teachers expressed concerns about the use of this technology if it negatively impacted teachers' workload. Thus, deployment of these technologies needs to be easy to implement, managed by teachers and cause no disruption to educational practices.

Teachers gave valuable and rich insights into potential underlying reasons behind patterns in physical activity data. Teachers' insights were categorized into six factors which have been previously identified in the Creating Active Schools Framework (Daly-Smith et al., 2020b). These findings demonstrate that the data encouraged teachers to think of school-based physical activity in a wider context, not just through traditional opportunities such as physical education. Teachers can use data to identify key school factors which may explain lower levels of physical activity and raises the possibility that wearable technologies could be used to facilitate conversations within schools to encourage key stakeholders to reflect on in-school physical activity.

The teachers acknowledged several factors which they believed explained some of the gender disparities. They felt girls' uniforms restricted their engagement in activities on the playground. These views are supported by Stanley and colleagues (Stanley et al., 2012) who ran focus groups with children aged 10–13 and reported that girls highlighted school uniform as a barrier to their physical activity. Teachers also spoke about gender stereotypes and innate physical differences creating barriers for girls' physical activity. It is reported that children are more likely to participate in activities which are aligned with their gender, however, many physical activities are perceived to align to masculine traits such as competitiveness, aggression, and stamina (Pawlowski et al., 2018; Looze et al., 2019; Peral-Suarez et al., 2020). Regular participation in activities will help develop the relevant skills, however, if girls are not engaging as regularly in physical activity, then perceptions of being physically disadvantaged may persist (Pawlowski et al., 2018). Teachers commented that girls had fewer physical activity opportunities and were potentially excluded from playground spaces because of the domination of football, for example. Several studies have highlighted that boys' football games dominate school playgrounds (Epstein et al., 2001; Martinez-Andres et al., 2017; Dudley et al., 2018; Spotswood et al., 2019; Peral-Suarez et al., 2020). There have been suggestions to consider how playground spaces are used, ensuring varied opportunities are available, particularly for girls (Dudley et al., 2018; Peral-Suarez et al., 2020). The findings from this study highlight the capacity for data from wearable technologies to facilitate these types of reflections on gender disparities, potentially providing a catalyst to help schools address these disparities.

Indeed, we demonstrate that teachers are likely to be able to readily access and reflect on data-driven insights when information is presented in the form of within-and between-school graphs for key physical activity comparisons and contrasts. As such, these findings support the notion that, instead of developing and testing standardized one-size-fits-all interventions, which have been limited in positively impacting children's activity to date (Love et al., 2018; Cassar et al., 2019), schools could use the data from wearables to inform and evaluate bespoke school-specific approaches to physical activity across the school day (Daly-Smith et al., 2020b).

Illustrative graphs of physical activity data were found to be user-friendly and understandable. However, some visualizations (i.e., bell curves and densely populated line graphs) were perceived to be more difficult to interpret than others. Since the use of data will be limited if communication is unclear (Hornik, 2002; Western et al., 2019), it will be important that future data communication strategies targeting teachers use graphs and visualizations that are sufficiently accessible and easy to understand. Some teachers showed an awareness of validity, reliability, and sensitivity concerns when using wearable technology. This is perhaps an important finding, highlighting some teachers may have a critical understanding of the data returned from these devices. Further research is needed in this area to understand how teachers respond to data depicting their own pupils' physical activity.

The data shown to teachers came from multiple UK schools, and it is possible that personalized school-specific data for each participating teacher may have prompted different responses. Teachers may have been more critical about (other) schools because of the lack of personalisation but, equally, reactions could have been stronger if they felt personally connected to data from their own schools. Teachers may also have been more confident explaining patterns in physical activity data if it was specific to their school. Future research should investigate the use of technology and data, and teacher interpretations and responses, within the context of interviewees' school settings. Such a study could also investigate if initial enthusiasm and motivation arising from in-school physical activity data leads to subsequent changes in teacher attitudes or practices.

Whilst efforts were made to recruit a diverse range of participants, teachers with a greater interest in physical activity were more likely to have participated due to the nature of the project and could be more enthusiastic than others about the potential use of wearable technologies. Furthermore, the use of digital communication technologies for the interviews may have excluded some participants who were apprehensive about using this software. It is also possible that interviewing in a global pandemic, which caused disruptions to normal practices, may have impacted teachers' priorities or outlooks relating to physical activity within schools.

## Conclusion

To our knowledge, this is one of the first studies to investigate the potential for physical activity data-driven insights to advance teachers' understanding and practices. Teachers were able to interpret the types of data produced from wearable technologies. Furthermore, findings suggest that primary school teachers in the UK are open to integrating wearable technologies for measuring children's physical activity into their practices. Visual representations of pupils' physical activity elicited strong responses from teachers and could therefore be used to inform teachers' behavior, school practices and policies ranging from whole-school changes to encourage physical activity, through to targeted approaches to tackle specific physical activity inequalities.

## Data Availability Statement

The raw data supporting the conclusions of this article will be made available by the authors, without undue reservation.

## Ethics Statement

The studies involving human participants were reviewed and approved by Research Ethics Approval Committee for Health at the University of Bath. The participants provided their written informed consent to participate in this study.

## Author Contributions

The study was designed by GKW, OP, and DT. Data was collected by GKW. Data preparation, analysis, and drafting of the paper was done by GKW and GW. All authors provided support for data interpretation, feedback on drafts, and approved the final manuscript.

## Funding

This research project did not receive any direct funding. GKW is funded by the Economic and Social Research Council (ESRC), training grant number ES/P000630/1.

## Conflict of Interest

DT declares a financial interest (shares) in the company which provided the source data used to develop the visualizations. Neither the company nor DT were involved in data analysis which was undertaken by GKW. The remaining authors declare that the research was conducted in the absence of any commercial or financial relationships that could be construed as a potential conflict of interest.

## Publisher's Note

All claims expressed in this article are solely those of the authors and do not necessarily represent those of their affiliated organizations, or those of the publisher, the editors and the reviewers. Any product that may be evaluated in this article, or claim that may be made by its manufacturer, is not guaranteed or endorsed by the publisher.

## Footnotes

^1^Moki Technology® (Moki Technology Ltd., 2021) is a wearable technology company which has designed a wrist-mounted accelerometer specifically for use within primary schools. Moki devices are commercially available, low-cost, and already being used and implemented at-scale by schools: to date (March 2021) Moki is being used in >500 schools (Moki Technology Ltd., 2021). The devices have no screen and the presentation of data to pupils is controlled by teachers. Moki devices estimate physical activity and return data on step count and moderate to vigorous physical activity (MVPA) averaged over 30-minute blocks, and devices connect to a bespoke software application via a contactless reader.
