# Supplementary material for: Teachers' Perspectives on the Acceptability and Feasibility of Wearable Technology to Inform School-Based Physical Activity Practices
Source: Front Sports Act Living. 2021 Nov 18;3:777105. doi: 10.3389/fspor.2021.777105 (PMC8636981; doi:10.3389/fspor.2021.777105)
Supplement: Supplementary file 1 [file Data_Sheet_1.docx]

Multi-Axis Simulation Table Testing

# Accelerometer

The Moki activity bands contains a triaxial accelerometer which is located within a silicone wristband (Moki Technology Limited- 11266496). Each unit is powered by a standard 3 Volt lithium CR-2032 battery, commonly used in watches. The accelerometers are scanned using a reader, which uses NFC technology and returns step count via a computer application. The devices have a 7-day activity data storage and a sampling frequency of 30-minutes. The accelerometers located within the bands are small circular disks with a 2.5cm diameter. 118 devices were subjected to testing in two controlled conditions. The accelerometers were removed from accompanying bands and double-sided floor tape (DS Scrim 306/250. Tape Range distributors Ltd, UK) was used to securely attach the bands to a Multi-Axis Stimulation Table alloy plate (see Figure 1). The devices were randomly positioned as they register movement in any direction. For this reason, the protocol was only run along the X-axis (longitudinal). The data from each device was downloaded using the accompanying Moki Technology software.

# Multi-Axis Simulation Table

Mechanical reliability testing was conducted on a Multi-Axis Simulation Table (MAST- 9720; Instron Structural Testing Systems Ltd., High Wycombe, UK) which is a 6 degree of freedom vibration test facility supplied by Instron Structural Testing Systems and is controlled by Instron 8800 electronics. The MAST- 9720 is powered via three vertical, one horizontal, and two lateral hydraulic actuators and is calibrated regularly to an accuracy of 0.1g (Figure 2). Only the horizontal actuator was used during this testing protocol. Before testing, a trial run was conducted without the accelerometers to ensure that the hydraulics were functioning at the optimum temperatures.

The frequency of oscillation and displacement amplitudes were used to replicate physiologically movements which tend to fall between 0.3 and 3.5Hz (Sun & Hill, 1993). The devices were shaken for two hours at a lower and higher frequency, allowing the devices to be subjected the same movements and the reliability to be investigated (2.5Hz and 3.5Hz). Protocol conditions can be viewed in Table 1. The testing conditions were inputted into the dedicated software (RS Replay, Instron Structural Testing Systems Ltd, High Wycombe, UK).

After completion of the testing, and when the MAST was safely parked, the accelerometers were removed, scanned, and the step counts downloaded using the accompanying Moki Technology software. An image of the MAST, device reader, and the software can be viewed in Figure 3. These values were recorded on Microsoft Excel in a comma-separated file format for analysis. The step counts were returned for the two, 2-hour testing periods.

# Results

Inter-device reliability is important when comparing multiple units (Metcalf et al., 2002). For each condition, the coefficient of variation (CV) was calculated as a measure of device reliability. Descriptive statistics are listed in Table 2. The mean step count for the protocol at 2.5Hz was 18000 ± 19 and for 3.5Hz 25197 ± 192 Individual units displayed low variability across all conditions with CV scores of 0.10% for 2.5Hz and 0.76% for 3.5Hz.

Bland and Altman plots were generated from all 118 devices for protocol 1 (Figure 4) and protocol 2 (Figure 5).

# Figures


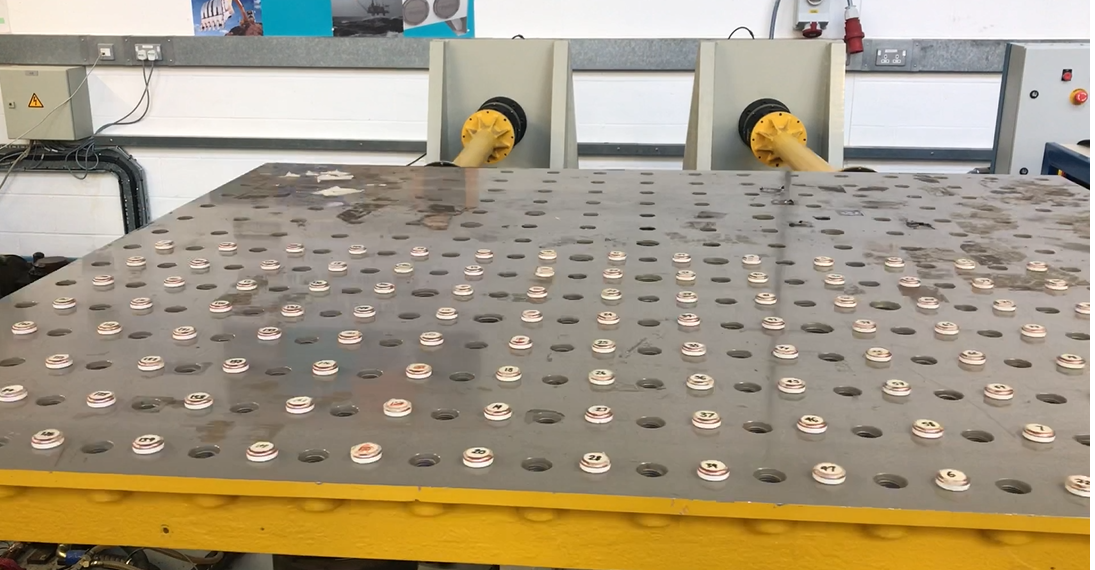


**Figure 1.** Accelerometers mounted to the alloy plate of the MAST


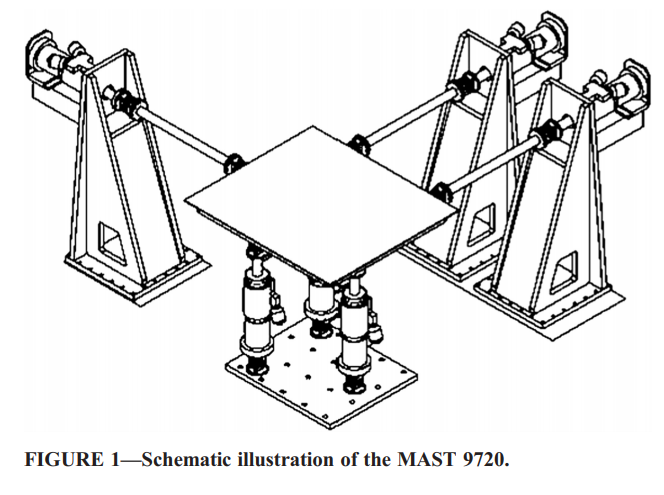


**Figure 2.** Schematic illustration of MAST 9720 taken from Horner et al 2011


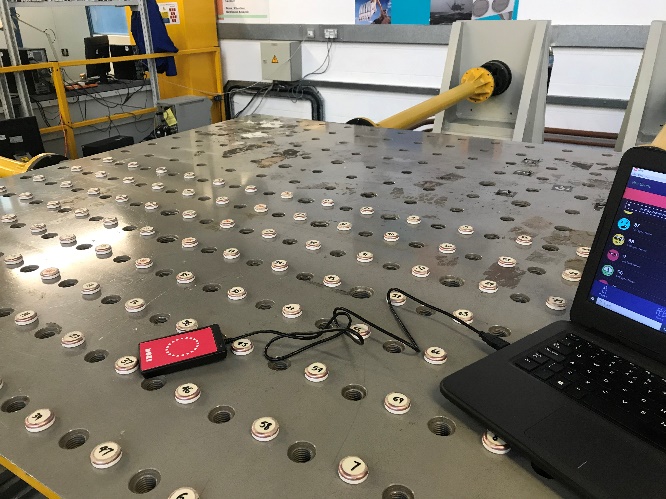


**Figure 3.** MAST Rig, accelerometers, device reader and Moki software

**Figure 4.** Protocol 1 (2.5Hz) Bland and Altman Plot

**Figure 5.** Protocol 2 (3.5Hz) Bland and Altman Plot

# Tables

Table 1. Protocol Conditions

| Condition | Duration (min) | Frequency (Hz) | Amplitude (mm) | Acceleration (g) | Velocity (mm/s) |
| --- | --- | --- | --- | --- | --- |
| 1 | 120 | 2.5 | 63 | 1.59 | 990 |
| 2 | 120 | 3.5 | 63 | 1.59 | 709 |

Table 2. Descriptive Statistics for both protocols

|  | Protocol 1 | Protocol 2 |
| --- | --- | --- |
| No. bands (N) | 118 | 118 |
| Minimum Steps | 17895 | 23222 |
| Maximum Steps | 18139 | 25224 |
| Mean Steps | 18000 | 25197 |
| SD Steps | 18.78 | 191.49 |
| CV (%) | 0.10 | 0.76 |

# References

Metcalf, B. S., Curnow, J. S., Evans, C., Voss, L. D., & Wilkin, T. J. (2002). Technical reliability of the CSA activity monitor: The EarlyBird Study. Medicine and Science in Sports and Exercise, 34(9), 1533-1537.

Sun, M., & Hill, J. (1993). A method for measuring mechanical work and work efficiency during human activities. Journal of biomechanics, 26(3), 229-241.
